# Associations of iron deficiency and depressive symptoms among young adult males and Females: NHANES 2017 to 2020

**DOI:** 10.1016/j.pmedr.2023.102549

**Published:** 2023-12-12

**Authors:** Cherry Y. Leung, Minjung Kyung

**Affiliations:** Department of Community Health Systems, School of Nursing, University of California San Francisco, San Francisco, CA, USA

**Keywords:** Iron deficiency, Iron status, Young adults, Depression, Mental health

## Abstract

Depression is one of the most prevalent mental health conditions throughout the lifespan. Notable differences in the prevalence of depression among females and males arise during adolescence and may peak during young adulthood. Since iron deficiency is a treatable condition that may contribute to depression, this topic among youth (18 to 25 years of age) needs to be further explored. Thus, our study examines the associations between three measures of iron (ferritin, serum iron, and transferrin saturation levels) with Patient Health Questionnaire-9 (PHQ-9) depressive symptoms and depression among young adult males and females using the National Health and Nutrition Examination Survey (NHANES) 2017–2020. Using multivariable Poisson and logistic regression models, adjusting for several demographic and clinical variables, we report 1) the prevalence of depression and 2) the associations between iron deficiency and depressive symptoms and depression among males and females. 917 participants were included in our study. More females (12.5 %) than males (6.8 %) had PHQ-9 depression. Males with ferritin (adjusted odds ratio [AOR] = 14.13, 95 % confidence interval [CI]: 1.51, 132.21), serum iron (AOR = 4.84, 95 % CI: 1.02, 22.92), and transferrin (AOR = 13.79, 95 % CI: 3.59, 53.06) deficiencies were at higher risk for depression, while females with ferritin deficiency (AOR = 0.34, 95 % CI: 0.11, 0.97) had a lower risk for depression. Our study highlights the need to focus on depression screening among young adults as well as risk factors for depression among this age group. Identifying risk factors and screening for iron deficiency, especially among females, should be considered as well.

## 1 Introduction

Depression is one of the most prevalent mental health problems among young adults (18–25 years of age). (National Institute of Mental Health, 2022) While the prevalence of depression among children is low without any sex differences, notable differences among females and males begin during adolescence and throughout young adulthood. (Yang et al., 2007) Additionally, depression continues to either persist or reappear during later life. (Mills et al., 2017) In the United States, an estimated 17 % of young adults had depression in 2020, with more females reporting depression (10.5 %) than males (6.2 %). (National Institute of Mental Health, 2022) Due to the poor mental and physical health outcomes (e.g., anxiety, illicit drug disorders, migraine, poor self-rated health, and increased work impairment) associated with depression, (Paradis et al., 2006) identifying potential modifiable risk factors, such as iron deficiency, are needed.

Iron deficiency, which is treatable, may contribute to depression, (Mills et al., 2017) yet there is limited research examining this association among young adults, a developmental period in which the brain is rapidly developing and individuals are becoming more autonomous therefore subject to environmental and lifestyle changes, including dietary changes. (Poobalan et al., 2014) Iron deficiency is a major cause of anemia, one of the most common nutritional disorders worldwide. (Kassebaum et al., 2014) Iron deficiency anemia is the impaired hemoglobin synthesis due to lack of iron, which can induce shortness of breath, dizziness, and fatigue. (Khedr et al., 2008) Iron also plays a critical role in brain development and the functioning of neurological, autoimmune, endocrine, and cardiovascular system, all of which can result in multiple health problems such as stroke, coronary heart disease, and endocrine disorders. (Khedr et al., 2008, Mirza et al., 2018) Additionally, many researchers have reported that iron deficiency is associated with poorer physical performance and lower work productivity of adults of all ages. (Khedr et al., 2008) However, the literature on the prevalence of iron deficiency among young adults in the United States and throughout the world is limited. These few studies focused on a large age range; one study reported that female adults 18 to 49 years of age have a higher prevalence of iron deficiency (33 %) compared to males (1.5 %) in Korea, (Kim et al., 2011) with similar findings reported in Belgium, Tehran and Japan. (Asakura et al., 2009, Shams et al., 2010, Pynaert et al., 2007).

Some growth periods require higher amounts of iron, especially those going through puberty or pregnancy, therefore individuals in these groups are more prone to iron deficiency. (Eicher-Miller et al., 2009) Specifically, many studies identified that women of reproductive age, typically defined as 12 to 49 years of age, thus including young adult females, is a risk factor for iron deficiency. (Sekhar et al., 2016) A study by Shariatpanaahi et al. (2007) analyzed ferritin levels (a measurement of iron levels) of 192 female medical students (24.5 years mean age) and reported that ferritin levels were significantly lower in students with depression compared to healthy students. (Vahdat Shariatpanaahi et al., 2007) Additionally, since young adults are now more independent and unsupervised, their diets may differ and they may be making poorer dietary choices, leading to an unbalanced diet and possibly deficiencies in key nutrients, vitamins, and minerals. (Winpenny et al., 2017).

While no studies have focused on the associations between iron and depression among this young adult age group, several studies have reported associations in adults, especially among older age adults 65 and above. Stewart and Hirani (2012) reported associations between higher serum transferrin receptor levels but not ferritin levels, both measurements of iron deficiency, with more depressive symptoms in older adults. (Stewart and Hirani, 2012) Similarly, Hosseini et al. (2018) reported that mean serum iron levels were lower in the depressive symptom group while there was no difference among ferritin levels between the groups. (Hosseini et al., 2018) Consistent with these findings, Su et al. (2016) reported no associations between ferritin levels and depressive symptoms among a cohort of Chinese adults. (Su et al., 2016) Studies used different measurements of iron deficiency among broader and older age groups, making findings difficult to compare, thus our goal is to determine whether a comprehensive set of measurements of iron status (ferritin, serum iron, and transferring) is associated with depression among this younger age group.

Due to the limited research on iron deficiency among young adults, as well as the associations between iron deficiency and depression, with known differences among males and females, our study aimed to describe the prevalence of these two important health outcomes among young adults, and stratified among males and females, as well as report on their associations using the National Health and Nutrition Examination Survey (NHANES) 2017 to 2020 dataset. We hypothesize that young adult females have both higher rates of iron deficiency and depression and while iron deficiency is associated with depression for both males and females, females with iron deficiency are at higher risk for depression. Research into iron deficiency, a modifiable risk factor of depressive symptoms and depression, is important since iron deficiency can both be prevented and also treated with increased iron-rich foods or over-the-counter supplements, (Semba, 2003) thereby reducing risk for depression.

## 2 Methods

### 2.1 Participants

Our study utilized the United States’ NHANES 2017 to 2020 dataset. The NHANES, conducted by the National Center for Health Statistics (NCHS) on 2-year cycles, assesses health and nutritional status of the United States population. The NHANES constitutes a nationally representative sample of non-institutionalized civilians selected by a stratified multistage probability sampling using phone interview and physical examination. The NHANES phone interview includes sociodemographic, dietary, and health related questions. At the end of the phone interview, a physical examination is scheduled at a NHANES Mobile Exam Center. The physical examination consists of medical, dental, and physiological measurements, as well as laboratory tests. We anticipated utilizing the NHANES 2017–2018 and 2019–2020 datasets. However, due to the COVID-19 pandemic, field operations were suspended in March 2020. Therefore, data from 2019 to March 2020 were used to represent the 2019–2020 dataset.

A total of 15,560 survey of all ages (birth to 80 years or more) were completed, with an interview response rate of 51 % and physical examination response rate of 46.9 %. Of these, there were 1,281 young adults aged 18 to 25 years. Since ferritin, a protein that stores iron, is one of the acute phase reactants accompanied by an inflammatory process, (Vahdat Shariatpanaahi et al., 2007) our study excluded 293 individuals who had inflammatory disorders, which were defined as having serum total folate less than 4 ng/mL (number; n = 4) or high-sensitive C-reactive protein more than 10 mg/L (n = 289). We also excluded pregnant individuals (n = 16). Fifty-five participants were excluded due to not having data on depression. Thus, 917 participants were included in our analysis. We received exemption for this study from the University Institutional Review Board since our study is a secondary analysis of the NHANES dataset.

### 2.2 Study variables and measures

#### 2.2.1 Exposures

Ferritin (μg/L), frozen serum iron (μg/L), and transferrin saturation (%) were measured as indicators of iron status. Ferritin is a protein that contains iron found in red blood cells and primarily used in evaluating iron metabolism and iron storage deficiency in the body. (Wick et al., 1995) Fresh serum or plasma were collected for analysis and measured by the Roche Cobas® e601 assay with total duration time of 18 min. After processing, ferritin specimens were stored at frozen temperature and protected from light. (National Health and Nutrition Examination Survey, n.d. a, National Health and Nutrition Examination Survey, n.d. b) Serum iron is circulating iron that is bound to transferrin (90 %) and serum ferritin (10 %). A three step process of the Roche method was used to measure frozen serum iron and transferrin saturation. (National Health and Nutrition Examination Survey, n.d. a, National Health and Nutrition Examination Survey, n.d. b) Specimens of frozen serum iron and transferrin saturation were stored at frozen or refrigerated temperatures, respectively. (National Health and Nutrition Examination Survey, n.d. a, National Health and Nutrition Examination Survey, n.d. b) Both frozen serum iron and transferrin saturation are useful in the measurement of iron deficiency along with ferritin, (National Health and Nutrition Examination Survey, n.d. a, National Health and Nutrition Examination Survey, n.d. b) therefore all three measurements were included in our study. Young adults who had ferritin levels of less than 30μg/L (Camaschella, 2015), frozen serum iron levels of less than 60μg/L, (Wu et al., 2002) or transferrin saturation level of less than 16 % (Camaschella, 2015) were defined as iron deficient.

#### 2.2.2 Outcomes

Depressive symptoms over the past two weeks were assessed using the nine-item Patient Health Questionnaire-9 (PHQ-9). (Spitzer et al., 1999) Depressive symptoms consist of: 1) having little interest in doing things, 2) feeling down, depressed, or hopeless; 3) trouble sleeping or sleeping too much; 4) feeling tired or having little energy; 5) poor appetite or overeating; 6) feeling bad about yourself; 7) trouble concentrating on things; 8) moving or speaking slowly or too fast; 9) thoughts you would be better off dead. The PHQ-9 scale has been validated, with good sensitivity (0.90) and specificity (0.99) in detecting depression in young adults. (Adewuya et al., 2006) Each response ranged from 0 for ‘not at all’ to 3 for ‘nearly every day,’ and a total score ranged from 0 to 27, with higher values indicative of more depressive symptoms. Depression was defined as having a PHQ-9 score of 10 or more based on the Diagnostic Statistical Manual of Mental Disorders, fourth edition criteria. (Adewuya et al., 2006).

#### 2.2.3 Confounders

Confounders included sex (male or female), age, race/ethnicity (non-Hispanic White, non-Hispanic Black, non-Hispanic Asian, Hispanic, and other), ratio of family income to poverty, mental health professional, body mass index (BMI) categories, physical activity (inadequate and adequate), dietary iron intake, and dietary supplements. The ratio of family income to poverty was an indicator of income established by the Department of Health and Human Services to represent the ratio of household income to the poverty guidelines, after controlling for family size and inflation. (U.S. Department of Health and Human Services, 2021) It was calculated by dividing family income by the poverty guidelines for the survey years and the score ranged from 1 to 5 with a lower score indicating lower socioeconomic status. BMI categories were defined as underweight (<18.5 kg/m^2^), healthy weight (18.5 to < 25 kg/m^2^), overweight (25 to < 30 kg/m^2^), and obese (>30 kg/m^2^). Physical activity was measured by the Global Physical Activity Questionnaire (GPAQ). (Armstrong and Bull, 2006) Moderate physical activity was defined as an activity that causes small increases in breathing or heart rate such as brisk walking or carrying light loads for at least 10 min continuously. Moderate physical activity also included going on a walk and using a bicycle. (Schuna et al., 2013) Vigorous physical activity was defined as an activity that causes large increases in breathing or heart rate like carrying or lifting heavy loads, digging or construction work for at least 10 min continuously. Respondents were asked to indicate the frequency (“In a typical week, on how many days do vigorous-intensity activities?”) and the duration of vigorous activity (“How much time spend doing vigorous-intensity?”) during work (e.g., paid or unpaid work, household chores, and yard work) and during recreational activities (e.g., sports, fitness, and recreational activities). In our study, total time spent in physical activity was calculated by multiplying the duration and frequency of both moderate and vigorous activity, where two minutes of vigorous activity was the equivalent to one minute of moderate activity. Based on the current physical activity guidelines, respondents were grouped as ‘inadequate’ (<150 min/week of physical activity) and 'adequate’ (>=150 min/week of physical activity). (U.S. Department of Health and Human Services, 2018) Mental health professional was derived from the question “Have you ever seen a mental health professional in the past 1 year?” with ‘yes’ and ‘no’ responses. Dietary information was obtained through two 24-hour dietary recall interviews, the first was in-person in the Mobile Examination Center and the second interview was by telephone 3 to 10 days later. Dietary iron (mg) intake was calculated from nutrients obtained from foods, beverages, and water. Our study calculated dietary iron intake based on the average intake across the two interviews; if the participant had only one record, then this record was used for analysis. Dietary supplements were derived from the question “Any dietary supplements taken in the past 24 h” with ‘yes’ and ‘no’ responses.

### 2.3 Data analysis

Descriptive statistics, such as frequencies, weighted percentages (%), weighted mean (m), and weighted standard deviations (SD), were used to describe the exposure, outcome, and demographic variables. We used weighted multivariable Poisson regression models to examine the associations between the three iron deficiency measures (ferritin, serum iron, and transferrin saturation) and depressive symptoms. We adjusted for characteristics such as age, sex, race/ethnicity, ratio of family income to poverty, mental health professional, BMI categories, physical activity, dietary iron intake, and dietary supplements. The results were reported as beta-coefficients (*β)* with 95 % confidence intervals (CIs).

Next, we used weighted multivariable logistic regression models to examine the associations between the three iron deficiency measures (ferritin, serum iron, and transferrin) and depression. We adjusted for characteristics such as age, sex, race/ethnicity, ratio of family income to poverty, mental health professional, BMI categories, physical activity, dietary iron intake, and dietary supplements. The results were reported as odds ratios (OR) with 95 % CIs.

Data was not transformed to correct for the distribution of the dependent variable, depressive symptoms, since we have a large dataset and linear regression is fairly robust to the violation of normality assumption with a large sample size. (Hoffman, 2003) Data analysis was performed using STATA version 16.0 (Stata Cooperation, College station, TX).

## 3 Results

Of the 987 young adults between 18 and 25 years of age, 917 had complete data on iron deficiency and PHQ-9 depressive symptoms and depression. The study sample characteristics and weighted prevalence of PHQ-9 depression by personal characteristics are shown in Table 1. Of the participants included in our study, 9.8 % reported depression, with a weighted prevalence of 9.5 % using the PHQ-9 survey. While there were more males (54 %) than females (46 %) in the sample, 13.5 % of females (12.5 % weighted) and 6.6 % of males (6.8 % weighted) had PHQ-9 depression. Using the three measures of iron deficiency, more depressed young adults, compared to non-depressed, had serum iron and transferrin deficiencies, although the differences were not statistically different. More obese participants were depressed (38 %) compared to not depressed (29 %). More depressed participants identified as having seen a mental health professional in the past year (31.4 %) compared to the non-depressed participants (12.1 %). Additionally, while there were no differences among dietary supplements among the depressed and non-depressed group, depressed participants had lower levels of iron intake through foods compared to the depressed group.

**Table 1:** Characteristics of young adults (18–25 years) by PHQ-9 depression from the 2017–2020 National Health and Nutrition Examination Survey (NHANES).

	Total (n = 917)	Depression (n = 90)	No Depression (n = 827)	
Participant characteristics	n (weighted %)	n (weighted %)	n (weighted %)	p-value
Iron deficiency				
Ferritin deficiency				
Yes (<30μ g/L)	181 (18.1)	19 (19.0)	162 (18.0)	0.730
No (≥30μ g/L)	736 (81.9)	71 (81.0)	665 (82.0)	
Serum iron deficiency				
Yes (<60 μg/L)	203 (20.7)	21 (25.7)	182 (20.2)	0.774
No (≥ 60μ g/L)	714 (79.3)	69 (74.3)	645 (79.8)	
Transferrin deficiency				
Yes (<16 %)	151 (15.2)	19 (24.2)	132 (14.2)	0.211
No (≥ 16 %)	766 (84.8)	71 (75.8)	695 (85.8)	
Age (Mean, SD)	21.1 (2.1)	21.3 (2.4)	21.1 (2.4)	0.483
Sex				
Male	486 (53.6)	32 (38.5)	454 (55.2)	**<0.001**
Female	431 (46.4)	58 (61.5)	373 (44.8)	
Race/ethnicity				
Non-Hispanic White	290 (55.4)	32 (58.5)	258 (55.1)	0.615
Non-Hispanic Black	214 (11.3)	17 (9.6)	197 (11.4)	
Non-Hispanic Asian	96 (5.1)	7 (4.3)	89 (5.2)	
Hispanic	254 (23.7)	26 (23.9)	228 (23.7)	
Other	63 (4.5)	8 (3.7)	55 (4.6)	
Ratio of family income to poverty,^a^ mean (SD)	2.1 (1.6)	2.2 (1.5)	2.1 (1.6)	0.743
BMI categories^b^				
Underweight (<18.5 kg/m^2^)	40 (4.0)	8 (5.4)	32 (3.8)	**0.002**
Healthy weight (18.5 to < 25 kg/m^2^)	381 (40.5)	24 (31.8)	357 (41.5)	
Overweight (25 to < 30 kg/m^2^)	226 (25.6)	21 (24.5)	205 (25.7)	
Obese (>30 kg/m^2^)	258 (29.9)	36 (38.2)	222 (29.0)	
Physical activity				
Inactive or insufficient (<150 min/week)	173 (16.3)	19 (21.0)	154 (15.8)	0.566
Sufficient (≥ 150 min/week)	744 (83.7)	71 (79.0)	673 (84.2)	
Mental health professional				
Yes	107 (13.9)	26 (31.4)	81 (12.1)	**<0.001**
No	810 (86.1)	64 (68.6)	746 (87.9)	
Dietary iron intake^b^, ^c^ (in mg), mean (SD)	13.3 (7.5)	11.3 (5.7)	13.6 (7.6)	**0.010**
Dietary supplements				
Yes	283 (33.8)	33 (42.9)	250 (32.8)	0.209
No	634 (66.2)	57 (57.1)	577 (67.2)	

Abbreviations: n, number; SD, standard deviation; BMI, body mass index, PHQ-9, Patient Health

Questionnaire-9; weighted %, weighted percentage.

a Range: 0–5 with higher values indicating higher socioeconomic status.

b Missing data: BMI categories (n = 12, 1.8 %), dietary iron intake (n = 41, 4.5 %).

c Range: 0.68–58.8 mg.

Supplementary Table S1 shows the characteristics of young adults by sex. There were more females that met the criteria for the three measurements of iron deficiency: ferritin (168 females, 13 males), serum iron (154 females, 49 males), and transferrin saturation (120 females, 22 males). The mean depressive symptom score for the sample was 3.5 (SD = 4.3), with males having a mean score of 2.9 (SD = 3.9) and females having a mean score of 4.1 (SD = 4.7). While more males were overweight or obese, more females were underweight. Lastly, more females engaged in sufficient physical activity, had less dietary iron intake, and took dietary supplements.

Table 2 shows that serum iron deficiency (*β* = 0.22, p-value < 0.01) and transferrin deficiency (*β* = 0.33, p-value < 0.001) were associated with more PHQ-9 depressive symptoms after adjusting for sex, age, race/ethnicity, ratio of family income to poverty, mental health professional, BMI, physical activity, dietary iron intake, and dietary supplements. In similarly adjusted models for males and females, only transferrin deficiency was associated with more depressive symptoms among males (*β* = 0.88, p-value < 0.001).

**Table 2:** Weighted associations of iron deficiency and PHQ-9 depressive symptoms of young adults (18–25 years) by sex: multivariable Poisson regression.

	Depressive symptoms					
	Overall^a^		Male^b^		Female^b^	
	β	95 % CIs	β	95 % CIs	β	95 % CIs
Iron deficiency (ref. No deficiency)						
Ferritin deficiency	0.03	−0.28, 0.34	0.89	−0.40, 2.18	−0.94	−1.92, 0.04
Serum iron deficiency	0.22*	0.02, 0.42	0.38	−0.26, 1.02	−0.51	−1.46, 0.44
Transferrin deficiency	0.33**	0.15; 0.50	0.88**	0.32, 1.44	−0.22	−1.00, 0.56

Abbreviations: PHQ-9, Patient Health Questionnaire-9; *β*, Beta coefficient; CIs, confidence intervals; ref., reference.

*p < 0.05, **p < 0.01, **p < 0.001.

a Adjusted for sex, age, race/ethnicity, ratio of family income to poverty, mental health professional, BMI, physical activity, dietary iron intake, and dietary supplements.

b Adjusted for age, race/ethnicity, ratio of family income to poverty, mental health professional, BMI, physical activity, dietary iron intake, and dietary supplements.

Table 3 shows the results of the associations of the three iron deficiency measurements with PHQ-9 depression while adjusting for sex, age, race/ethnicity, ratio of family income to poverty, mental health professional, BMI, physical activity, dietary iron intake, and dietary supplements. There were no associations in the overall models with all young adults (both males and females). In the adjusted model for males, serum iron deficiency (OR = 4.84, p-value < 0.01) and transferrin deficiency (OR = 13.79, p-value < 0.001), compared to no deficiency, were associated with a higher odds of PHQ-9 depression. In the adjusted model for females, compared to no ferritin deficiency was associated with a lower odds of PHQ-9 depression (OR = 0.34, p-value < 0.01).

**Table 3:** Weighted adjusted associations of iron deficiency and PHQ-9 depression of young adults (18–25 years) by sex: multivariable logistic regression.

	Depression					
	Overall^a^		Male^b^		Female^b^	
	OR	95 % CIs	OR	95 % CIs	OR	95 % CIs
Iron deficiency (ref. No deficiency)						
Ferritin deficiency	0.80	0.33;1.92	14.13*	1.51, 132.21	0.34*	0.11, 0.97
Serum iron deficiency	1.32	0.51; 3.40	4.84*	1.02, 22.92	0.51	0.17, 1.57
Transferrin deficiency	1.69	0.79; 3.61	13.79**	3.59, 53.06	0.72	0.29, 1.81

Abbreviations: PHQ-9, Patient Health Questionnaire-9; OR, odds ratio; CIs, confidence intervals; ref., reference.

*p < 0.05, **p < 0.01, **p < 0.001.

a Adjusted for sex, age, race/ethnicity, ratio of family income to poverty, mental health professional, BMI, physical activity, dietary iron intake, and dietary supplements.

b Adjusted for age, race/ethnicity, ratio of family income to poverty, mental health professional, BMI, physical activity, dietary iron intake, and dietary supplements.

## 4 Discussion

To the best of our knowledge, this is the first study to include three measurements of iron deficiency to examine their associations with young adult depressive symptoms and depression as well as examine sex differences. Our study showed that about 9.5 % of young adults reported at least moderate depression (PHQ-9 score >=10) in this NHANES 2017–2020 cohort. Mean depressive symptoms scores were low, not meeting the threshold for mild depression (PHQ-9 score >= 5), (Kroenke et al., 2001) although the range of depressive symptom scores varied from 0 to 27. Additionally, more females reported having PHQ-9 depression and having more depressive symptoms. Overall, among all three measurements of iron, more females had ferritin, serum iron, and transferrin saturation deficiencies. While both serum iron deficiency and transferrin deficiency were associated with a higher risk of PHQ-9 depressive symptoms for young adults, only males with transferrin deficiency had a higher PHQ-9 depression score compared to no deficiency, with a 0.88 point difference in scores. On the other hand, while there were no associations between the iron measures and PHQ-9 depression for young adults as a whole, males with ferritin, serum iron, and transferrin deficiencies, compared to no deficiency, were at 14.13, 4.84, and 13.79 times higher odds of having PHQ-9 depression, respectively, whereas females with ferritin deficiency had a lower odds (OR = 0.34) of PHQ-9 depression.

Our findings may be an underestimation of the prevalence of depression (9.5 %) in this age group, which is less than the estimated 17 % of young adults who reported having a depressive episode in the past year according to the National Institutes of Mental Health (NIMH) in 2020. (National Institute of Mental Health, 2022) While the PHQ-9 questionnaire is well-validated among several populations, including this young adult population, (Adewuya et al., 2006, Spitzer et al., 1999) reporting bias may still exist due to the sensitive nature of mental and emotional health questions. Additionally, the measurement of depression in our study was self-reported, whereas a clinician diagnosis is often preferred. Nonetheless, in large epidemiological studies, clinician diagnosis and report are not feasible. Further, information on mental health professionals indicated that non-depressed participants saw a mental health professional in the past year, indicating the possibility that depressive symptoms may be under good control, thus the PHQ-9 questionnaire did not fully capture these participants since the questionnaire asks about symptoms within the past two weeks. Nonetheless, depression screening with a validated tool, such as the PHQ-9, should be considered at primary care settings due to ease of administration and scoring as well as per the recommendation of the US Preventive Services Task Force. (U.S. Preventive Services Task Force, 2023)

Consistent with the literature regarding sex differences, (Herreen et al., 2022, Salk et al., 2017) the prevalence of depression among young adult females (12.5 %) was nearly double that of young adult males (6.8 %), highlighting the need to determine and clarify risk factors, whether, biological, psychological or social, associated with depression stratified by sex. Similarly, females compared to males in this sample had higher rates of iron deficiency, supporting that females during the reproductive age have lower iron levels likely due to menstruation, especially when females have heavy menstrual bleeding. (Munro et al., 2023) Other conditions affecting iron levels include pregnancy and childbirth, but our study excluded pregnant women.

Our findings that males with transferrin deficiency had higher depressive symptom scores and males with ferritin, serum iron, and transferrin deficiency had a higher risk of depression are similar to findings of Yi et al. (2011). (Yi et al., 2011) Yi and colleagues reported no significant associations among women (mean age 41.6) but found that men (mean age 44.2) with lower serum ferritin concentrations had more depressive symptoms. (Yi et al., 2011) On the other hand, other studies consisting of pre-menopausal women (Hunt and Penland, 1999) and older adults 65–83 years of age (Baune et al., 2006) reported no significant associations between iron deficiency and depression. Of the few studies examining depressive symptoms, the age of participants ranged from young adults to middle or older adults, making comparisons difficult. Nonetheless, similar to our findings, but with a focus on females, Noorazar et al.’s (2015) study examining depressive symptoms among females with a Major Depressive Disorder reported no associations between serum ferritin deficiency and depressive symptoms. (Noorazar et al., 2015) Our study found that, contrary to the literature, (Vahdat Shariatpanaahi et al., 2007) females with ferritin deficiency had a lower risk of depression. However, our findings with gender differences may have been due to chance since there were a small number of females with PHQ-9 depression as well as a small number of males meeting criteria for both iron deficiency and PHQ-9 depression.

Our findings with the association between the three measures of iron deficiency and depression may differ due to the different functions of ferritin, serum iron, and transferrin. Per our findings, it is a possibility that ferritin had the most influence on depression when stratified by sex due to our significant associations among both males and females. While the underlying mechanisms are still unknown, iron may play a role in neurotransmission, where iron has been hypothesized to synthesize the neurotransmitters dopamine, serotonin, and norepinephrine, all of which have been implicated in depression. (Berthou et al., 2022) Iron has also been implicated in cytokine-mediated neuroinflammation, which leads to the dysregulation of neurotransmitters. (Berthou et al., 2022).

While our study has many strengths, it nonetheless has limitations. First, our study was cross-sectional therefore does not allow for causal inference. Second, our study consisted of participants with few depressive symptoms and a lower prevalence of depression compared to the NIMH estimates; (National Institute of Mental Health, 2022) therefore, our study findings cannot be generalized to participants with more severe depressive symptoms. However, the PHQ-9 questionnaire provides us with information on symptomology. While the participants did not meet criteria for moderate depression or mild depression, most participants had some symptoms, reiterating the need to screen and follow participants closely at the primary care level. Third, our study relied on self-reports which can lead to recall and desirability bias, which may influence our findings. Fourth, although our study excluded those with inflammation based on CRP levels and controlled for several confounders, residual confounding may still exist. We cannot preclude that all participants with inflammatory disorders, such as metabolic syndrome and diabetes mellitus, were excluded. Fifth, we neither excluded nor adjusted for participants who were on anti-depressant medications since only the 2017–2018 dataset, but not the 2019–2020 dataset, had this information. Nonetheless, only one participant reported anti-depressant use. While we adjusted for dietary supplements where 34 % of our adolescents reported taking supplements, we did not have any information on iron supplements. Including nuanced information about supplements and diet is necessary for the research, management, and treatment of iron deficiency as well as further research on depression. Sixth, our significant findings with wide ORs and 95 % CIs may be inflated due to the small sample of participants with depression and in each of the iron deficiency groups, thus additional research focused on those with depression and stratified by sex should be considered.

## 5 Conclusion

Our study provides evidence that iron deficiency is associated with young adult depression when stratified by sex. Our study findings indicate that young adult females with ferritin deficiency have lower odds of depression while young adult males with ferritin, serum iron, and transferrin deficiencies have higher odds of depression. Additionally, we found that young adult females have a higher prevalence of both iron deficiency and depression. It is crucial that future studies examine depression stratified by sex and the differing risk factors, including biological, behavioral, and social determinants. Additionally, our study highlights the need for more resources allocated to young adults, especially females, to identify both iron deficiency and other modifiable nutritional risk factors, including supplement intake, related to depression, as iron deficiency can be prevented with adequate nutritional intake. Lastly, young adults regardless of low iron levels should be considered for depression screening.

## Declaration of competing interest

The authors declare that they have no known competing financial interests or personal relationships that could have appeared to influence the work reported in this paper.

## Data Availability

I have shared the link to the NHANES dataset.
