# Supplementary material for: Associations of iron deficiency and depressive symptoms among young adult males and Females: NHANES 2017 to 2020
Source: Prev Med Rep. 2023 Dec 12;37:102549. doi: 10.1016/j.pmedr.2023.102549 (PMC10788288; doi:10.1016/j.pmedr.2023.102549)
Supplement: Supplementary data 1 [file mmc1.docx]

Supplementary Table 1. Characteristics of young adults (18-25 years) by sex from the 2017-2020 National Health and Nutrition Examination Survey (NHANES)

|  | Male  (n=486) | Female  (n=431) |  |
| --- | --- | --- | --- |
| Personal characteristics | n (weighted %) | n (weighted %) | p-value |
| Iron deficiency |  |  |  |
| Ferritin deficiency |  |  |  |
| Yes (<30$\mu$g/L) | 13 (2.5) | 168 (36.0) | **<.001** |
| No ($\geq30\mu$g/L) | 473 (97.5) | 263 (64.0) |  |
| Serum iron deficiency |  |  |  |
| Yes (<60$\mu g/L$) | 49 (9.5) | 154 (33.6) | **<.001** |
| No ($\geq$60$\mu$g/L) | 437 (90.5) | 277 (66.4) |  |
| Transferrin deficiency |  |  |  |
| Yes (<16%) | 22 (4.1) | 129 (28.0) | **<.001** |
| No ($\geq$16%) | 464 (95.9) | 302 (72.0) |  |
| Age (Mean, SD) | 21.0 (2.4) | 21.2 (2.4) | 0.360 |
| Race/ethnicity |  |  |  |
| Non-Hispanic White | 148 (55.8) | 142 (54.9) | 0.341 |
| Non-Hispanic Black | 108 (10.6) | 106 (12.1) |  |
| Non-Hispanic Asian | 57 (5.2) | 39 (5.1) |  |
| Hispanic | 143 (25.0) | 111 (22.2) |  |
| Other | 30 (3.5) | 33 (5.7) |  |
| Ratio of family income to poverty,^a^ mean (SD) | 2.2 (1.6) | 2.1 (1.5) |  |
| BMI categories^b^ |  |  |  |
| Underweight (<18.5kg/m^2^) | 14 (2.0) | 26 (6.2) | **0.023** |
| Healthy weight (18.5 to <25kg/m^2^) | 198 (36.7) | 183 (44.9) |  |
| Overweight (25 to <30kg/m^2^) | 135 (28.4) | 91 (22.4) |  |
| Obese (>30kg/m^2^) | 135 (32.9) | 123 (26.5) |  |
| Physical activity |  |  |  |
| Inactive or insufficient (<150 min/week) | 60 (12.3) | 113 (21.0) | **<.001** |
| Sufficient ($\geq$150 min/week) | 426 (87.7) | 318 (79.0) |  |
| Mental health professional |  |  |  |
| Yes | 52 (14.2) | 55 (13.7) | 0.332 |
| No | 434 (85.8) | 376 (86.3) |  |
| Dietary iron intake^b,c^ (in mg), mean (SD) | 14.86 (8.4) | 11.7 (6.0) | **0.013** |
| Dietary supplements |  |  |  |
| Yes | 116 (27.8) | 167 (40.6) | **<.001** |
| No | 370 (72.2) | 264 (59.4) |  |
| PHQ-9 Depression |  |  |  |
| Yes | 32 (6.8) | 58 (12.5) | **<.001** |
| No | 454 (93.2) | 373 (87.5) |  |

Abbreviations: n, number; SD, standard deviation; BMI, body mass index, PHQ-9, Patient Health

Questionnaire-9; weighted %, weighted percentage

^a^Range: 0-5 with higher values indicating higher socioeconomic status

^b^Missing data: BMI categories (n=12, 1.8%), dietary iron intake (n=41, 4.5%)

^c^Range: 0.68-58.8mg
